# Should We Worry About the Inter-Implant Gap in the Tibia? A Finite Element Analysis of Revision TKA and Distal Plating

**DOI:** 10.3390/medicina62030450

**Published:** 2026-02-27

**Authors:** Renato Caravellos Glória, Pedro José Labronici, Anderson Freitas, Vincenzo Giordano

**Affiliations:** 1 Departamento de Ortopedia e Traumatologia, Universidade Federal Fluminense (UFF), Niterói 24070-090, RJ, Brazil; 2 Serviço de Ortopedia e Traumatologia Prof. Nova Monteiro, Hospital Municipal Miguel Couto, Rio de Janeiro 22430-160, RJ, Brazil; 3 Hospital Ortopédico e Medicina Especializada (HOME), Brasília 70200-730, DF, Brazil; 4 Clínica São Vicente, Rio de Janeiro 22451-100, RJ, Brazil

**Keywords:** periprosthetic tibia fracture, inter-implant fracture, total knee arthroplasty, revision TKA, finite element analysis, locking plate, stress distribution

## Abstract

*Background and Objectives*: The management of periprosthetic tibial fractures distal to revision Total Knee Arthroplasty (TKA) presents a biomechanical challenge, often requiring extramedullary locking plates when long stems preclude nailing. While in femoral fractures the gap between the stem and plate is a well-documented stress riser, requiring implant overlap to prevent an inter-implant fracture, this specific biomechanical scenario has not been studied in the tibia, and it remains unclear if the femoral dogma of mandatory overlap applies to the straight, centrically loaded tibial anatomy. This study utilized Finite Element Analysis (FEA) to evaluate stress distribution in the tibial inter-implant gap. *Materials and Methods:* A comparative FEA was performed using a validated standardized tibia model simulating a healed distal fracture. Two cemented revision TKA constructs (50 mm and 80 mm stems) were modeled. These were paired with medial locking plates of varying lengths (10, 12, and 14 holes) to create different inter-implant distances. Eight distinct configurations, including non-plated controls, were subjected to physiological axial compression and three-point bending. Outcome measures included von Mises stress and total displacement. *Results:* The analysis revealed no significant stress concentration in the bone within the inter-implant zone across all plated models, regardless of the gap size. Instead, the addition of plates universally reduced bone stress compared to controls, effectively transferring load to the fixation hardware. Peak stresses were consistently observed in the proximal locking screws rather than the bone gap. The longest plates (14 holes) offered superior construct rigidity and stress distribution. *Conclusions*: Under the conditions evaluated in this preclinical finite element model, the tibia does not exhibit a biomechanical requirement for implant overlap to prevent stress risers. Our findings suggest that extramedullary fixation with the longest available anatomical locking plate represents a biomechanically plausible strategy for these fractures, even if an inter-implant gap remains.

## 1. Introduction

The demand for primary total knee arthroplasty (TKA) is rapidly increasing, driven by an aging population with higher activity levels. Projections estimate that 3.5 million primary TKAs will be performed annually in the United States by 2030 [1]. This trend is accompanied by a proportional rise in the number of revision arthroplasties and associated complications, including extensor mechanism failure, periprosthetic joint infection, and periprosthetic fractures [2,3]. Femoral periprosthetic fractures are the most common type of fractures around a total knee arthroplasty, with periprosthetic tibial fractures representing approximately 0.4–0.5% [4]. However, despite being a rare event, the management of tibial fractures distal to a TKA, particularly those in the diaphyseal or distal metadiaphyseal region, is complex. And periprosthetic tibial fractures are associated with high complication (38%) and reoperation (28%) rates [5].

Although intramedullary nailing is currently the standard-of-care treatment for native tibial fractures, its use is often challenging in periprosthetic tibial fracture either due to a very short proximal fragment or the presence of the tibial prosthetic component. The latter is even more critical in tibial revision components, where long, press-fit diaphyseal-engaging stems are frequently used, making an antegrade nailing approach virtually impossible [6,7]. Consequently, extramedullary fixation with locking plates has become a primary treatment modality particularly for these injuries with revision tibial stems [8].

In the case of very distal fractures, the use of a plate can create a challenging biomechanical scenario, with a bone segment situated between two rigid implants, which theoretically generates a stress concentration. In the femur, this inter-implant gap has been shown, in some studies, to create a significant stress riser, increasing the risk of subsequent fracture [9,10,11]. This phenomenon is largely attributed to the anatomy of the femur, specifically its natural anterior and lateral bowing, as well as the offset between its mechanical and anatomical axes, which induce substantial bending moments under axial loading. However, the tibia possesses fundamentally different biomechanical characteristics. It is a relatively straight bone, and its mechanical and anatomical axes are nearly collinear and aligned with the vertical load vector during stance. This anatomical distinction suggests that the tibia primarily experiences compressive forces rather than the complex bending moments seen in the femur. In fact, the mean peak bending moments in the frontal plane are greater in the femur (71–130 Nm) than in the tibia (26 to 43 Nm), while the mean peak femoral compression forces (1330–1936 N in the mid-diaphysis) correspond to about half of the tibial compression (2299–5224 N) [12].

Based on these key biomechanical and anatomical differences, we hypothesized that, unlike in the femur, the construct of a locking plate and a revision TKA stem would not create a significant stress concentration in the inter-implant tibial segment. To test this hypothesis, we used a finite element method (FEM), a computational tool for analysing complex structural problems in orthopaedics [13,14]. Therefore, the objective of this study was to qualitatively and quantitatively analyse the stress distribution in the inter-implant bone gap using the FEM to specifically evaluate displacement and von Mises equivalent stress (VM) in virtual models of a plated distal tibia fracture below a revision TKA component under axial and bending loads, after complete union of the experimental fracture.

## 2. Materials and Methods

### 2.1. Study Design

A biomechanical experimental study was conducted using a FEM. Eight virtual models were created and analysed. The models included two control groups, consisting of a tibia with only a revision TKA cemented tibial component, using either a 50 mm or 80 mm stem (“Prosthesis 50” and “Prosthesis 80”, respectively). The six study groups combined these same two revision stems with three different lengths of pre-contoured anatomical medial locking plates: 10-hole, 12-hole, and 14-hole.

The simulations were designed to represent the biomechanical environment of a healed (united) distal tibia fracture (AO/OTA 43) that had been treated with a locking plate in a patient with a pre-existing ipsilateral revision TKA.

### 2.2. Finite Element Model Generation

The geometric models for both the bone and the implants were developed utilizing Rhinoceros™ 6 software (Robert McNeel & Associates, Seattle, WA, USA). For the osseous structure, a validated, public domain “Standardized Tibia” virtual 3D model was employed [15]. Derived from CT scans of a Sawbones^®^ artificial tibia (Pacific Research Labs, Vashon Island, WA, USA), this model features a total length of 367 mm, a proximal articular surface measuring 66 mm mediolaterally and 42 mm anteroposteriorly, a cortical thickness of 4 mm, and a mean intramedullary diameter of 12 mm. Its reliability in previous FEM studies has been well-documented [16,17].

Solid 3D models of the cemented Attune^®^ Revision Knee System Fixed Bearing (DePuy Synthes^®^, J&J Company, Warsaw, IN, USA) were generated featuring 50 mm and 80 mm stem extensions. The specific dimensions for the tibial baseplate and stem diameters were selected from the manufacturer’s available options to ensure the optimal fit within the Standardized Tibia model (Table 1). Furthermore, 3D models representing the internal fixation hardware were created based on the LCP^®^ 3.5 mm medial tibial locking plates (DePuy Synthes^®^) in lengths of 10, 12, and 14 holes, utilizing 3.5 mm locking screws modelled according to the manufacturer’s specifications.

### 2.3. Model Assembly and Meshing

The virtual surgical assembly and subsequent analysis were performed using SimLab™ 2024 software (Altair Engineering, Inc., Troy, MI, USA) within the HyperWorks platform, utilizing theOptistruct 2024 solver on a desktop computer (AMD Ryzen 7 5700X 8-Core 3.40 GHz, 32 GB RAM, Windows 11 64-bit). The OptiStruct solver is a commercially benchmarked finite element solver.

**Figure 1:**
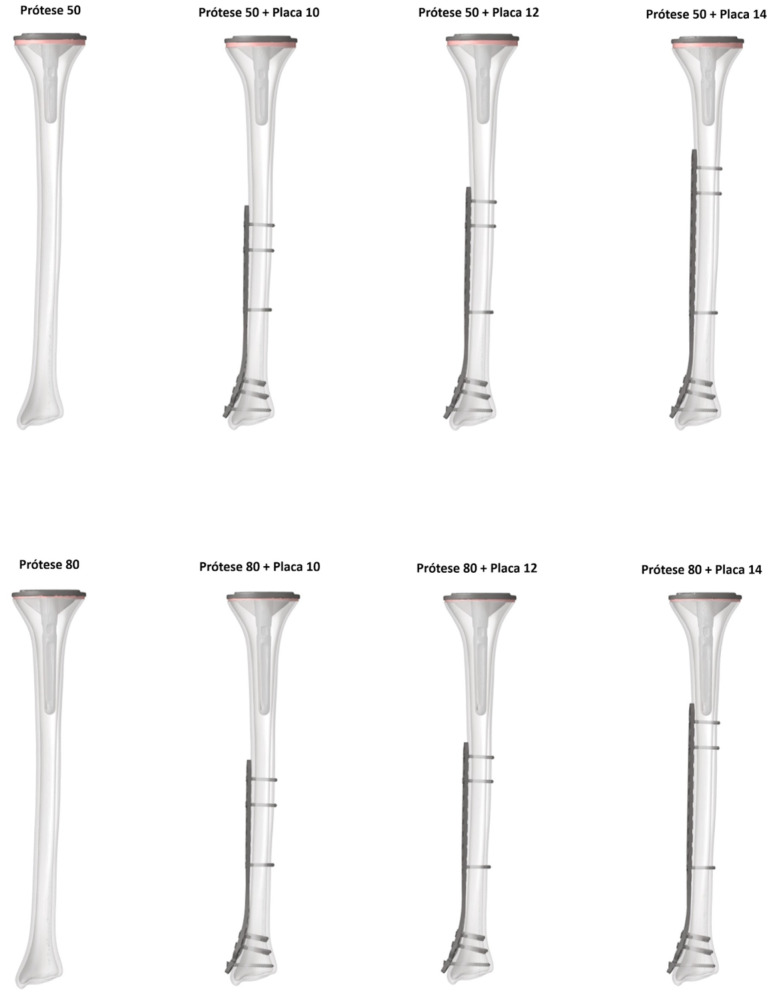
Models obtained for the finite element analysis.

The prosthetic components were virtually implanted according to the manufacturer’s surgical technique. A proximal tibial resection was made 10 mm below the articular surface, perpendicular to the mechanical and anatomical axes in the coronal plane and with a 3° posterior slope in the sagittal plane. A 1 mm cement mantle was simulated around the stem and on the underside of the baseplate. The LCP plates were positioned anatomically on the medial tibial surface. For all plate models, the five most distal screw holes (including the malleolar hole) were filled. Proximally, screws were placed in the first and third holes from the end of the plate, as well as the hole closest to the working area. All screws were modelled as fixed-angle (locking) constructs. This resulted in 8 total configurations for analysis (2 control and 6 study models), with distances between implants ranging from 72.73 mm to −11.26 mm (overlap) in the plated models, as shown in Figure 1 and Table 2.

The geometric domains were discretized using second-order tetrahedral elements. An average element edge length of 1.0 mm was used for cortical and cancellous bone and 0.5 mm for the implants. The mesh was refined in contact regions to an average edge length of 0.8 mm. A mesh convergence test confirmed that these dimensions achieved mesh independence, with no significant changes in peak stress values.

### 2.4. Material Properties and Interfaces

All materials (bone and implants) were modelled as linear elastic, isotropic, and homogeneous. The material properties based on values from established literature [16,18,19] are listed in Table 3.

The interfaces between screw-plate, screw-bone, and cement-bone were considered bonded. Frictional contacts were defined for the bone–plate interface (coefficient of friction, µ = 0.37) and the cement–prosthesis interface (µ = 0.25), as reported in previous studies [17,20,21]. These interface properties are summarized in Table 4.

### 2.5. Boundary and Loading Conditions

#### 2.5.1. Axial Compression

To simulate physiological loading, the tibia’s mechanical and anatomical axes were aligned with the vertical (Z) axis. An axial compressive load of 1663 N was applied to the proximal articular surface, corresponding to approximately 1.5 times the body weight of a 75 kg adult during the mid-stance phase of gait [22]. The load was distributed 60% through the medial plateau and 40% through the lateral plateau. The distal articular surface of the tibia was fixed in all six degrees of freedom.

#### 2.5.2. Three-Point Bending

To simulate bending forces, the tibia was positioned horizontally along the (Y) axis. The proximal and distal ends of the model were fixed. A 500 N load was applied in the anteroposterior direction over a 25 mm area at the exact midpoint of the tibial diaphysis [23,24].

#### 2.5.3. Outcome Measures

For each model and loading condition, total displacement (mm) and von Mises equivalent stress (MPa) in the implants and bone were measured and analysed.

## 3. Results

### 3.1. Axial Compression Loading

#### 3.1.1. Von Mises Stress Analysis

Under axial loading, no stress concentration occurred in the inter-implant bone gap in any of the plated models. The introduction of a locking plate resulted in a notable redistribution of stress, substantially decreasing stress in the bone and prosthesis while transferring the load to the plate and screws.

**Figure 2:**
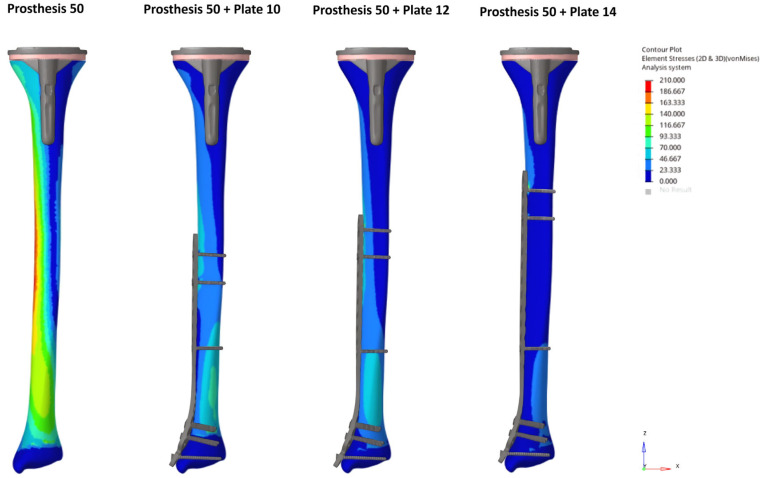
Bone von Mises stress distribution under axial loading for the Prosthesis 50 control model and constructs with 10, 12, and 14-hole plates. Note the progressive reduction in stress concentrations as plate length increases, including within the inter-implant gap.

**Figure 3:**
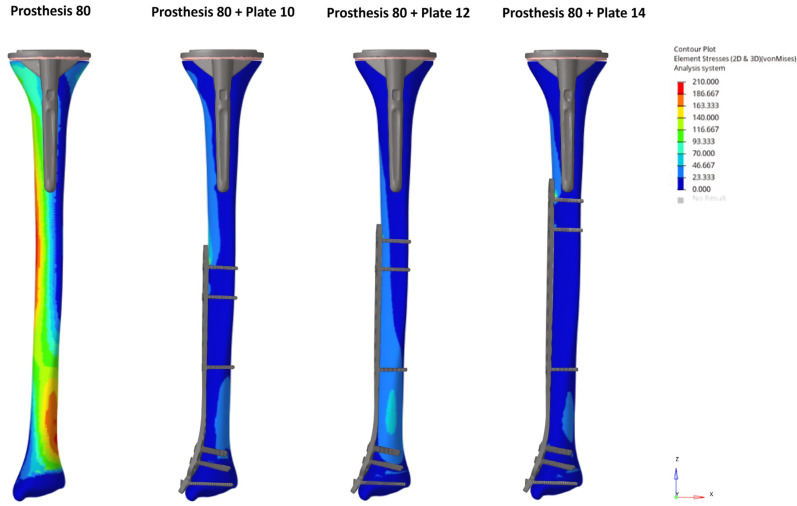
Bone von Mises stress distribution under axial loading for the Prosthesis 80 control model and constructs with 10, 12, and 14-hole plates. Note the progressive reduction in stress concentrations as plate length increases, including within the inter-implant gap.

The unplated Prosthesis 50 control model exhibited the highest bone stress (204.9 MPa). The addition of any plate reduced this peak stress by more than half, a reduction that was observed throughout the bone, including within the inter-implant gap. The Prosthesis 80 models demonstrated inherently lower bone stresses than their 50 mm counterparts, a trend that continued with the addition of plates (Figure 2 and Figure 3).

**Figure 4:**
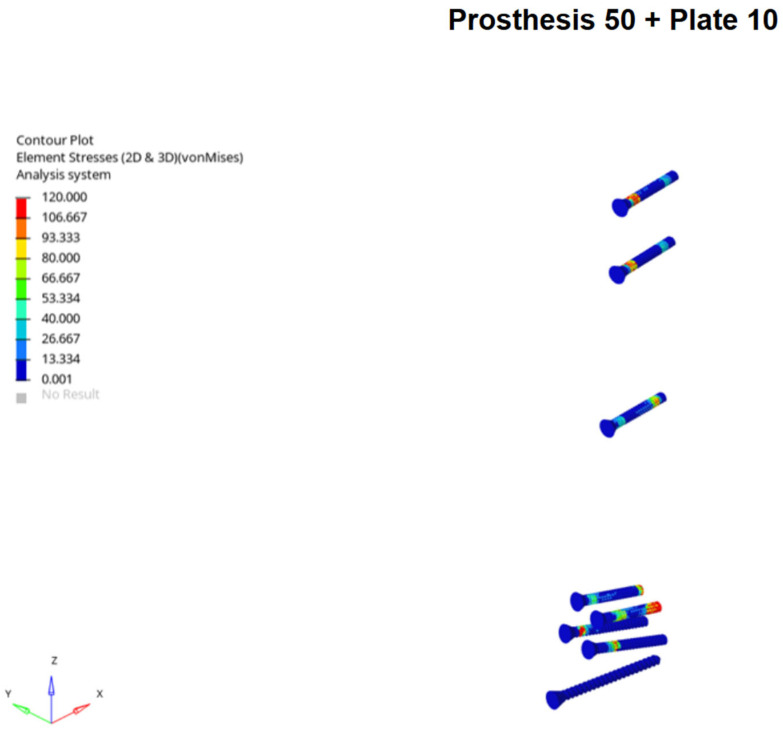
Example of screws Von Mises stress distribution under axial loading for Prosthesis 50 associated with 10-hole plate models, showing stress concentration on the screw located nearest to the inter-implant gap.

**Figure 5:**
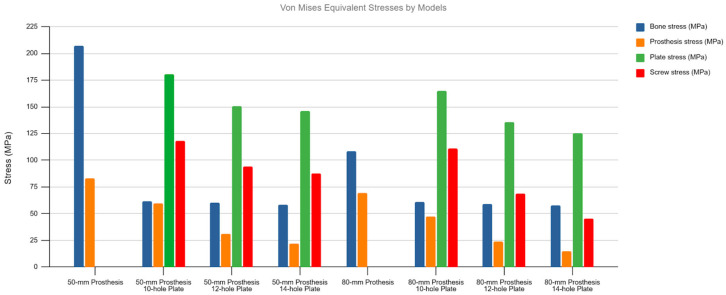
Von Mises stress values for all studied models under axial loading. Observe the overall trend of decreasing stress across all components as both plate and stem lengths increase.

Conversely, the highest stresses in the implants were seen in the plated models, particularly in the Prosthesis 50 + Plate 10 configuration. A progressive reduction in stress on both plates and screws was observed as plate length increased, a trend that was more pronounced in the Prosthesis 80 models. Notably, the screws located nearest to the inter-implant gap consistently exhibited the highest stress concentrations. Stress on the prosthetic components was highest in the unplated control models and was progressively reduced with the addition of longer plates. The Prosthesis 80 + Plate 14 combination demonstrated the lowest overall stress levels across all components (Figure 4 and Figure 5).

#### 3.1.2. Displacement Analysis

The addition of plates significantly reduced total displacement, indicating a marked increase in structural stiffness across the entire construct, including the inter-implant bone segment.

**Figure 6:**
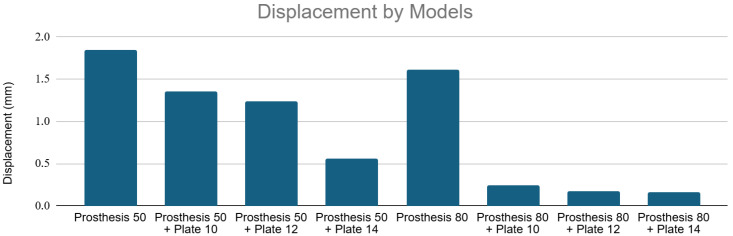
Displacement values by models under axial loading. Note the significant reduction in total displacement with the addition of increasingly longer plates and stems.

The Prosthesis 50 control model exhibited the greatest displacement (~1.85 mm). Adding a 10, 12, and 14-hole plate progressively reduced this displacement to a minimum of ~0.6 mm (Prosthesis 50 + Plate 14). The Prosthesis 80 control model was inherently stiffer (~1.6 mm displacement). Plating further reduced displacement to below 0.25 mm, with the Prosthesis 80 + Plate 14 configuration proving to be the most rigid construct tested (~0.2 mm displacement). Figure 6 shows displacement values for all models under axial loading.

### 3.2. Three-Point Bending Loading

#### 3.2.1. Von Mises Stress Analysis

Consistent with the axial loading results, no stress concentration was observed in the inter-implant bone gap under three-point bending. The addition of plates resulted in a modest reduction in bone stress while transferring considerable load to the plate and screws.

**Figure 7:**
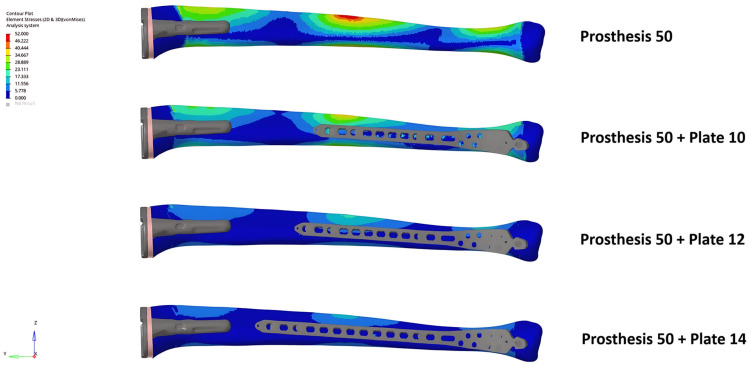
Bone von Mises stress distribution under three-point bending for the Prosthesis 50 control model and constructs with 10, 12, and 14-hole plates. Note the progressive reduction in stress concentrations as plate length increases, including within the inter-implant gap.

**Figure 8:**
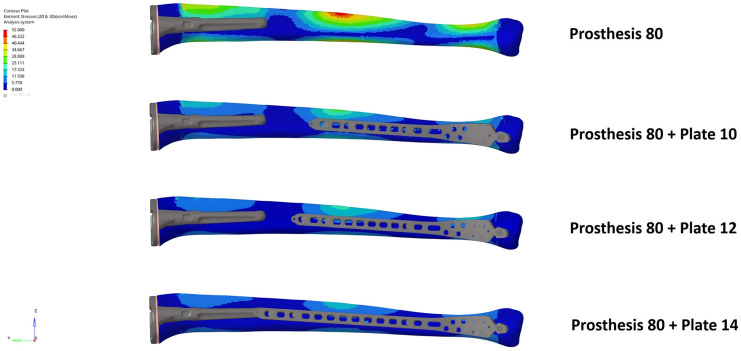
Bone von Mises stress distribution under three-point bending for the Prosthesis 80 control model and constructs with 10, 12, and 14-hole plates. Note the progressive reduction in stress concentrations as plate length increases, including within the inter-implant gap.

**Figure 9:**
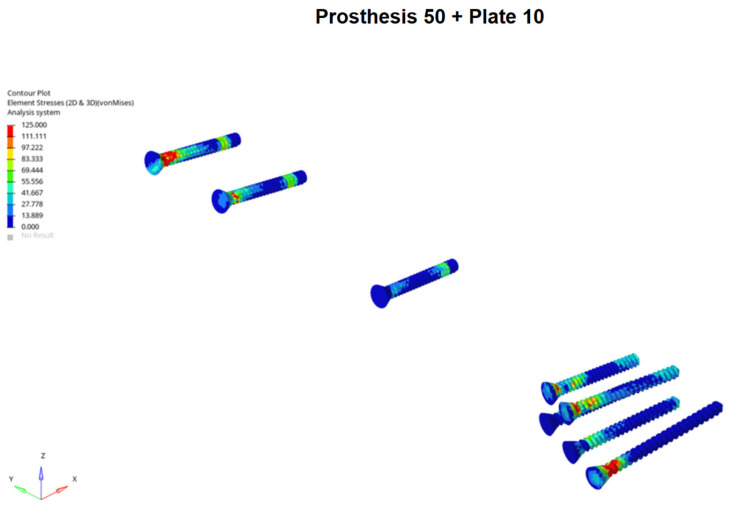
Example of screws Von Mises stress distribution under three-point bending for Prosthesis 50 associated with 10-hole plate models, showing stress concentration on the screw located nearest to the inter-implant gap.

**Figure 10:**
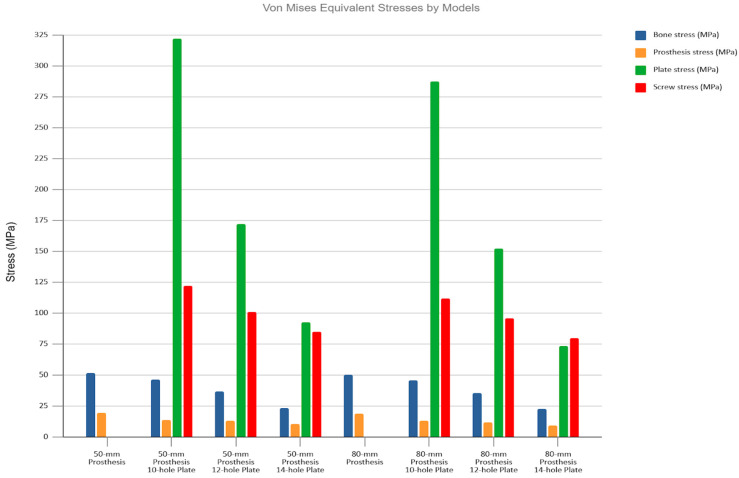
Von Mises stress values for all studied models under three-point bending. Observe the overall trend of decreasing stress across all components as both plate and stem lengths increase.

The highest bone stresses (~52 MPa) were recorded in the two control models. Plating slightly decreased these values, including in the inter-implant gap. The Prosthesis 80 models consistently showed lower bone stress than the equivalent Prosthesis 50 models. Similar to the axial loading scenario, the screws positioned closest to the inter-implant gap were found to bear the highest stresses (Figure 7, Figure 8, Figure 9 and Figure 10).

#### 3.2.2. Displacement Analysis

**Figure 11:**
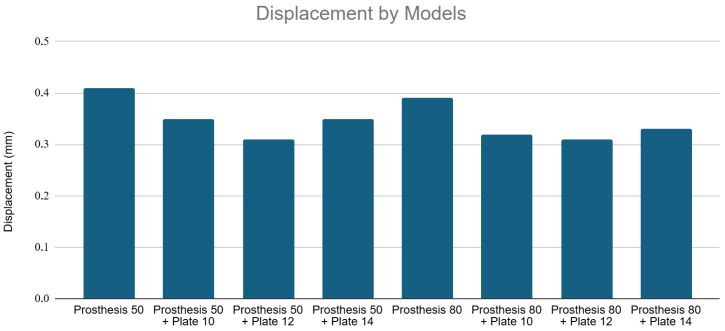
Displacement by models under three-point bending. Note that while longer stems intrinsically increased stiffness, the 12-hole plate constructs demonstrated the maximum structural rigidity under bending loads, exhibiting less displacement than the longer 14-hole configurations.

The Prosthesis 80 models were intrinsically stiffer under bending loads, showing less displacement than the corresponding Prosthesis 50 models. The Prosthesis 50 control model showed the highest displacement (~0.41 mm). The 12-hole plate constructs (Prosthesis 50 + Plate 12 at ~0.31 mm and Prosthesis 80 + Plate 12 at ~0.305 mm) proved to be the most rigid configurations, exhibiting less displacement than their 14-hole counterparts. Figure 11 shows displacement values for all models under three-point bending.

## 4. Discussion

Contrary to concerns based on femoral biomechanics [25,26], using FEM, our study showed that the inter-implant bone segment between a revision TKA stem and a distal tibial locking plate does not become a significant stress riser. In all simulated models, the addition of a plate improved the overall stress distribution and increased the construct’s structural stiffness compared to the prosthesis-only controls. This suggests that the primary biomechanical effect of plating in this scenario is protective.

The high stresses observed in the locking screws nearest the inter-implant gap, particularly with shorter plates, may represent the successful neutralization of forces that would otherwise have concentrated in the bone. The load is effectively transferred from the bone to the implant construct. The observation that these screw stresses decreased as the inter-implant gap was reduced (by using longer stems or longer plates) suggests a potential protective effect of minimizing the gap length.

Although there is a paucity of biomechanical data regarding the tibial inter-implant gap, our findings stand in contrast to some femoral studies where the inter-implant gap is a known risk factor for fracture. Walcher et al. [27] analysed 38 synthetic femoral models with stem-to-plate distances ranging from an 8 cm gap to a 6 cm overlap. They observed that the group with a 4 cm inter-implant distance exhibited significantly higher stresses and a lower load to failure (2684 N) compared to the “distant” group (3713 N), suggesting a higher risk of fracture with smaller gaps. In a biomechanical analysis of composite femora with inter-prosthetic gaps ranging from 0 to 20 cm, Quirynen et al. [28] observed that implant proximity significantly influenced failure strength. The authors found that the 0 cm gap configuration yielded the highest mean fracture load (9415 N), which was significantly higher than all other gap sizes. Conversely, the lowest fracture load was observed in the larger 15 cm gap models (7346 N).

These discrepancies might be explained by the fundamental biomechanical differences between the two bones. The femur, with its anterolateral bow and valgus alignment of the knee, is subjected to significant bending moments under axial load. The tibia, being a relatively straight bone with collinear mechanical and anatomical axes, primarily experiences compressive forces [12]. This anatomical distinction is the most likely reason that a stress riser did not manifest in our tibial models.

From a clinical standpoint, these results are highly relevant. Surgeons face a practical challenge, as the longest commercially available anatomical distal tibial plates (~14 holes) may be insufficient to overlap a long revision TKA stem. While longer, non-anatomical plates exist, their design is poorly suited for obtaining adequate multi-screw fixation in the distal metaphyseal fragment, which may be a problem, especially in osteoporotic bone. Our findings suggest that while overlapping the implants may be ideal in the case of femoral periprosthetic fractures [28], in the scenario of a periprosthetic fracture of the distal tibia, surgeons can likely use the longest available anatomical plate without creating a dangerous stress riser in the gap between the implants. From a biomechanical perspective, the fixation strategy should therefore prioritize achieving a stable construct based on fracture pattern and bone quality, regardless of whether a gap or overlap exists between the implants.

The primary strength of this study is its originality. To the best of our knowledge, this is the first FEM analysis to investigate the biomechanics of this specific and increasingly common clinical scenario in the tibia after a TKA. By using a validated standardized tibia model and basing material properties and loading conditions on established literature, we have created a reproducible and clinically relevant simulation. Our results undoubtedly highlight a subject that is still little explored but is gradually being discussed more in the literature, not only because periprosthetic tibial fractures after TKAs are increasing, but mainly because the complications are much greater than those observed after periprosthetic femoral fractures [5]. Furthermore, our results show that the use of double plating in the treatment of these fractures, theoretically biologically detrimental, may not be necessary, since no increase in stress concentration between the implants was observed. Several authors have used double-plating strategies due to the lack of a sufficiently long implant to extend beyond the tip of the tibial stem [29,30].

The study is not without limitations. First, as a computational model, it lacks validation from a clinical trial or a mechanical benchtop experiment. Second, we did not model the influence of soft tissue attachments, though their contribution to axial and bending loads is likely less significant in the tibia than in the femur. Third, our model represents an idealized scenario and does not account for anatomical variations between patients or the wide variety of implant designs available. Fourth, we did not use plates with more than 14 holes, which may be available from other companies. However, our findings showed that these may not be necessary since there was no noticeable increase in inter-implant stress concentration. Fifth, we did not evaluate the biomechanical behavior of lateral plates. Although the lateral surface of the distal tibia provides better soft tissue coverage—reducing the risk of hardware prominence and wound complications—medial plating remains the preferred technique for distal tibial osteosynthesis [31]. This preference is driven by both biological and mechanical factors. Biologically, the periosteal blood supply to the anterior two-thirds of the tibial shaft is mainly delivered through the lateral surface [32]; thus, medial plating is less disruptive to local perfusion, which is a critical concern in compromised environments such as the periprosthetic region. Mechanically, medial plates have demonstrated superior stability compared to anterolateral plates, particularly in varus fracture patterns [33]. Consequently, our finite element model simulated the most common clinical scenario. Sixth, our computational model assumed homogeneous bone properties and simulated a fully healed fracture state with an intact fibula. Consequently, it does not account for the biological and time-dependent realities frequently encountered in compromised periprosthetic bone. Specifically, our static model did not evaluate the effects of cyclic fatigue loading, delayed union or non-union, bone remodeling and stress shielding, osteolysis around the tibial revision stem, or screw loosening over time. Ignoring these critical factors inherently limits the direct clinical applicability of our findings to these complex conditions. Furthermore, by assuming an intact tibia-fibula complex, we did not evaluate the altered load-sharing present in acute fractures with associated fibular fractures. It is important to emphasize, however, from a clinical perspective, that additional fibular fixation does not significantly reduce the rate of varus–valgus or anteroposterior malreduction in distal metaphyseal tibial fractures; it is primarily recommended in the presence of an associated syndesmotic injury to prevent rotational deformity [34,35]. Seventh, our simulation focused on axial and bending forces, excluding torsional loads. Although the tibia is a bone where the mechanical and anatomical axes coincide, torsional forces, along with complex physiological scenarios such as stair ascent/descent, stumble loading with high peak forces, and repeated cyclic fatigue, can significantly contribute to periprosthetic fracture patterns by increasing local stress concentrations. However, it is known that axial impact loading is the most common type of load causing a tibial fracture and that off-axis loading, generated by bending loads, contributes to this [36,37,38]. Thus, we prioritized the loading modes most prevalent in biomechanical studies. Consequently, it is crucial to acknowledge that our model evaluates static structural stiffness rather than dynamic failure risk. Therefore, our findings suggest a mechanical tendency rather than a definitive clinical recommendation. Further clinical and biomechanical studies are warranted to validate our findings under other experimental scenarios.

## 5. Conclusions

This finite element study demonstrated that the inter-implant bone segment between a revision TKA and a distal tibial locking plate is not an area of notable stress concentration under the simulated static axial and bending loads. The addition of a plate universally increased construct stiffness and improved stress distribution in the bone. Our findings suggest that, in a preclinical FEA model, extramedullary fixation with the longest available anatomical locking plate demonstrates favorable biomechanical behavior, even when an inter-implant gap remains. However, these results reflect a mechanical tendency and should be interpreted with caution until validated by future clinical studies.

## Figures and Tables

**Table 1:** Dimensions of the tibial prosthetic components. (AP: anteroposterior; ML: mediolateral).

Dimensions (mm)	50 mm Stem	80 mm Stem
AP baseplate	42.8	42.8
ML baseplate	65	65
Baseplate thickness	4.1	4.1
Proximal stem diameter	14	14
Distal stem diameter	14	10
Stem length	50	80
Total implant length	75.3	105.3

**Table 2:** Distances between implants in the plated models.

Model	Distance (mm)
Prosthesis 50 + Plate 10	72.73
Prosthesis 50 + Plate 12	56.79
Prosthesis 50 + Plate 14	21.43
Prosthesis 80 + Plate 10	40.19
Prosthesis 80 + Plate 12	24.25
Prosthesis 80 + Plate 14	−11.26

**Table 3:** Biomechanical properties of the materials used in the simulation.

Material	Young’s Modulus (GPa)	Poisson’s Ratio
Osteoporotic cortical bone	14.2	0.3
Osteoporotic cancellous bone	0.104	0.3
Plate (Steel)	200	0.3
Prosthesis (Titanium)	110	0.3
Cement (PMMA)	2.28	0.3

**Table 4:** Interface properties used in the models.

Interface	Type/Coefficient of Friction (µ)
Bone-Plate	Frictional (µ = 0.37)
Screw-Plate	Fixed (Bonded)
Screw-Bone	Fixed (Bonded)
Cement-Bone	Fixed (Bonded)
Cement-Prosthesis	Frictional (µ = 0.25)

## Data Availability

The raw data supporting the conclusions of this article will be made available by the authors on request.

## Abbreviations

The following abbreviations are used in this manuscript:

TKA	Total knee arthroplasty
FEM	Finite element method
VM	Von Mises equivalent stress
